# Effects of biopreparations based on *Bacillus* and *Trichoderma*, combined with mineral and organic fertilization and a *Pisum sativum* L. forecrop on improving the tolerance of Maize plants to drought stress

**DOI:** 10.1371/journal.pone.0322718

**Published:** 2025-05-06

**Authors:** Dominika Radzikowska-Kujawska, Tomasz Piechota, Karolina Jarzyniak, Przemysław Łukasz Kowalczewski, Piotr Wojewódzki

**Affiliations:** 1 Department of Agronomy, Poznań University of Life Sciences, Poznań, Poland; 2 Department of Biochemistry and Biotechnology, Poznań University of Life Sciences, Poznań, Poland; 3 Department of Food Technology of Plant Origin, Poznań University of Life Sciences, Poznań, Poland; 4 Department of Biogeochemistry and Soil Science, University of Science and Technology in Bydgoszcz, Bydgoszcz, Poland; Graphic Era Institute of Technology: Graphic Era Deemed to be University, INDIA

## Abstract

The increased frequency of extreme weather phenomena, such as heat waves and drought, adversely affects the condition of plants. The need to strive for more sustainable methods of growing plants requires undertaking researches that focus on strengthening the immunity of plants using methods that have a positive impact on both crops and the natural environment. The aim of the study was to assess the effectiveness and compare the effects of selected microbiological preparations based on *Bacillus* bacteria and *Trichoderma* symbiotic fungi, combined with mineral (NPK) and organic fertilization (manure) and a *Pisum sativum* L. forecrop on improving the tolerance of maize plants to drought stress. The pot experiment was carried in 2023 as a two-factor experiment in three replicates. Physiological parameters were assessed based on measurements of photosynthetic efficiency (A - CO_2_ assimilation rate, E - Transpiration Rate, Gs – Stomatal Conductance) and chlorophyll content (CCI) and fluorescence (F0 - initial fluorescence, Fm - maximum fluorescence, Fv/Fm - maximum photochemical efficiency of PSII, Yield - quantum yield of the photochemical reaction in PSII, ETR – electron transport rate, NPQ - Non - Photo-chemical Quenching), as well as soil respiration (NCER- Net CO_2_ Exchange Rate, W flux- Net H_2_O Exchange Rate, Ce- Soil Respiration) and biometric measurements (dry mass of shoots and roots).The measurement of photosynthesis efficiency under drought stress clearly indicated the highest, significant effect caused by Trichoderma preparation with both fertilizers. In the control, CO_2_ assimilation was practically inhibited due to drought (98% drop), while in the plants in which the Trichoderma preparation was used together with half dose of NPK and manure, there was only a slight decrease (1% and 13% respectively). A greatest, significant improvement in the DM of roots under drought was noted in plants in which the Pisum forecrop was applied together with NPK and manure (230% and 168% respectively). Pisum forecrop and treatments with microbiological preparation containing *Trichoderma*, make it possible to reduce the fertilization dose by at least half. This is particularly important in view of the global trend of increasing drought stress and efforts to improve soil quality.

## Introduction

Since its domestication, estimated at 9,000 years ago, maize (*Zea mays* L.) has been playing an increasingly important and multifaceted role in global agri-food system [1]. Grain maize is grown on about 205 million hectares worldwide per year, making it the most widely grown crop after wheat. However, when annual grain maize production is considered in the context of the yield, which is 1,174 million tonnes (Mt), it ranks first and exceeds rice and wheat production by half [2]. The main reasons for the constant growth of interest in this species around the world are its versatile use, relative ease of cultivation and adaptation to climatic conditions. At the global level, maize is used as feed (56% of production), 20% for non-food uses, and 13% for food [2]. In recent years, the possibility of using this species for the production of renewable energy, mainly for the production of biogas, has also become increasingly important [3]. Thanks to its genetic diversity, maize shows an extraordinary ability to adapt to different climatic conditions [4]. Additionally, maize ability to function well in a wide range of tropical, subtropical and temperate environments is due to its excellent photosynthetic efficiency (C4) [1]. It is worth noting that maize landraces play a crucial role in climate change adaptation as a reservoir of genetic diversity that can be harnessed for breeding stress-tolerant cultivars [4].

Climate-related events threaten all dimensions of the food supply chain, and consequently, food and nutrition security [5–6]. The European Environment Agency [7] indicates the main climate change consequences related to water resources are increases in temperature, shifts in precipitation patterns and snow cover, and a possible increase in the frequency of flooding and droughts. The main effect of drought stress and the usually accompanying high temperature is increased evaporation of water from the ground, resulting in a significant reduction in the inflow of rainwater to the roots [8]. Long-term water deficiency increases the likelihood of photosystem (PSII) damage, which is the cause of reduced photosynthetic efficiency and increased dissipation of absorbed energy in the form of non-photochemical quenching [9]. As a result, the proper course of physiological processes is disturbed, which contributes to the inhibition of their growth and development, which ultimately results in lower yields [10]. According to the assumptions of the European Green Deal (European Commission, 2019), in order to counteract further environmental degradation, a policy was introduced aimed at, among others, reducing the use of plant protection products (PPP) in favor of biological methods. The most pro-environmental solutions promoted include the cultivation of species from the legume family as forecrops or catch crops in order to improve the physical and biological properties of the soil. It is also pointed out that it is necessary to take care of biodiversity both in the context of proper crop rotation and the biodiversity of soil fauna - naturally occurring strains of soil bacteria and symbiotic fungi. Both bacteria and fungi living in the soil increase the availability of nutrients for plants. Akinsemolu [11] indicates enhancing agricultural productivity, reducing reliance on pesticides and herbicides, and restoring optimum soil composition are all positive outcomes of green microbiology that promote environmental sustainability. Among natural methods of plant protection, the application of microbiological preparations supporting plant cultivation is becoming more widely used. Especially plant growth promoting mediums produced on the basis of fungi and bacteria appear in the agromarket. Many of these products are made from *Trichoderm*a spp. fungi and *Bacillus* spp. bacteria.

*Trichoderma* comprises a genus of phylamentous fungi, saprophytic, avirulent and opportunistic plant symbionts, inhabiting mainly the soil [12]. Members of the *Trichoderma* genus are ubiquitous organisms of different ecosystems in a wide range of climatic zones [13]. For their positive effects on cultivated plants these fungi have been widely studied and commercially marked as biofungicides, biofertilizers and soil amendments [14]. *Trichoderma* spp. exert beneficial effect to the agriculture such as increase in plants photosynthetic capability and yields, efficient absorption of nutrients and abiotic as well as biotic stress tolerance [15–17]. Harmann et al. [18] indicated that *Trichoderma* increased root development and crop yield, the proliferation of secondary roots, and seedling fresh weight and foliar area. The other beneficial effect of *Trichoderma* is production of auxins which could stimulate root development and plant growth [19]. Vargas et al. [20] demonstrated that *T. virens* colonization of maize rhizosphere induced higher photosynthetic rates and systemic increases in the CO_2_ uptake in leaves. The genus *Trichoderma* spp. is also characterized by mycoparasitic activity, i.e. it parasitizes other fungi that may constitute potential plant pathogens [21–22]. The researches [23–24] indicated that that *T. harzianum* from semi-arid soils may be employed to improve maize plants’ tolerance to water stress. It was revealed that maize seedling colonization by *T. harzianum* enhanced systems of antioxidative enzymes and promote maize germination. Additionally, it has been revealed that the *Trichoderma* spp. strain has the ability to physiologically protect plants against oxidative damage by reducing the accumulation of lipid peroxidases (oxidative stress indicators). The *Trichoderma harzianum* T22, used in experiment, include both mycoparasitic, competitive and synergistic activities in relation to the plant, supporting its development and nutrient uptake, enabling the use of this fungus as an effective agent in the fight against drought stress [25].

Also *Bacillus* spp., particularly *B. velezensis* is considered as a significant advance in the manufacturing of various biocontrol products [26]. *B. velezensis* is a gram-positive, endospore-forming bacteria isolated from the mouth of the river Vélez in Málaga (southern Spain) [27]. The research of Chen et al. [28] revealed that *B. velezensis* can produce a variety of hydrolases (lignocellulose, cellulose, and hemicellulose), can effectively improve plant protein feed utilization and nutritional quality. In addition, the *Bacillus velezensis* strain unlocks phosphorus in the soil and provides the plant with atmospheric nitrogen. Additionally, they are common inhabitants of the microflora of plant root systems, which means that their impact on the composition of microbial communities there is insignificant. Some bacteria are able to form biofilms in the rizosphere which promote the growth of plants, protecting them against pathogens by secreting antimicrobial compounds [29–31]. These bacteria also increase plant tolerance to the adverse effects of abiotic stresses, such as heat waves and drought [32]. The research of Tiepo et al. [33] concerning influence of *B. velezensis* on maize indicated that inoculated plants were characterized with increased roots and shoots dry weight, greater level of chlorophyll fluorescence and lipid peroxidation as well as with anatomical changes - increase in the vascular cylinder area.

High soil quality means its ability to maintain plant productivity, which is particularly important under conditions of environmental stress. Soil quality is evaluate based on properties like, the enzymatic activity, the contents of micro- and macro-elements, the contents of C and N, humidity, the pH, the soil structure and texture. The population numbers and activities of the soil microorganisms as well as fabaceae forecrop and organic fertilization have a positive effect on all those properties [34]. Organic fertilization as well as fabaceae fore- or cover crop is also an important aspect in the process of increasing the biodiversity of soil organisms and the level of humus in the soil. Deliberate actions to improve soil quality in a broad sense are intended to increase the productivity of plants and improve their adaptation to water shortage conditions [35]. Using organic fertilizers and planning a fore- or cover crop in the form of plants from the fabaceae family does not significantly increase production costs, but may have a significant impact on its volume and quality in the long term.

The aim of the research undertaken was to assess the effectiveness and compare the effect of selected microbiological preparations based on strains of soil bacteria of the genus *Bacillus* and symbiotic fungi of the genus *Trichoderma*, combined with mineral and organic fertilization and a *Pisum sativum* L. forecrop on improving the tolerance of maize plants to drought stress. The effect of microbiological preparations on plants yield is widely examined, however there is no much evidence on the plants response to drought stress taking into account the effect of different kind of fertilization and forecrop combined with microbiological preparations employment. We hypothesized that the Pisum forecrop and the use of different fertilizers (mineral vs. organic) would affect the effectiveness of microbiological preparations and the nutritional status of plants and, consequently, their adaptive abilities to drought stress. We also hypothesized that microbiological preparations as well as Pisum forecrop will allow for a reduction in the dose of fertilizers, which will be beneficial both economically and ecologically.

## Materials and methods

### Microbiological preparations

As the experimental factors there were tested two microbiological preparations: fungal agent Trianum-P and bacterial agent Biomega^TM^.

The Trianum-P is commercially available biological fungicide produced by Koppert company (Denmark). The physical form of the preparation are water-soluble granules, containing live spores of the fungus *Trichoderma harzianum* T-22 at a concentration of 1x10⁸ cfu/gram (according to the manufacturer’s label).

Biostimulator Biomega™ is a liquid preparation contains strains of *Bacillus velezensis* bacteria produced by SMP Agro Sp. z o.o. (Poland).

The doses of particular preparations are indicated in Table 3.

**Table 3:** Characteristics and doses of fertilizers and preparations used.

No.	Fertilization combination	Dose/pot	Producer
1.	N 34% full dose	2.00 g	Anwil S.A.
2.	P_2_O_5_ 19% full dose	3.60 g	Siarkopol
3.	K_2_O 51% full dose	0.95 g	SoluKem
4.	cattle manure full dose (2.8% N: 2.8% P_2_O_5_: 2.0% K_2_O)	24.3 g	Florovit
5.	Trianum P (*Trichoderma harzianum* T-22 1x10⁸ cfu/gram)	0.012 g	Koppert
6.	Biomega TM (*Bacillus velezensis*)	0.004 ml	SMP Agro sp. z o.o.

### Growing condition

The pot experiment was carried out in the garden (the period from sowing seeds to the induction of drought stress), as well as in the greenhouse (during drought stress) and phytotron (during physiological measurements) that belongs to the Department of Agronomy at the University of Life Sciences (Poznań, Poland, 52.4327, 16.9004). The experiment was carried out in 2023 as a two-factor experiment with four replications. For each of the 12 factor’s levels, for both watered and drought-stressed plants, 4 pots were set up, giving a total of 96 pots. At each stage of the experiment, in the garden and in the greenhouse, the pots were randomly moved to eliminate environmental differences. The first-order research factor was drought stress, with the following levels: Control (volumetric soil moisture: 23 ± 3%); Drought (volumetric soil moisture: 3 ± 1%). The second-order research factor included the tested combinations of fertilization, preparation application and forecrop, with the levels as in Table 1. The maize plants (*Zea mays* L.) used in the experiment was a medium-late, stay green variety called ‘Farmoritz’. The seed was purchased directly from the producer (Farmsaat).

**Table 1:** Levels of the second-order research factor - combinations of fertilization, preparation and forecrop.

No.	Factor	Treatment - method of use
1.	Control	without the use of preparations, forecrop and fertilizers
2.	NPK	mineral fertilizer - full dose of N, P, K
3.	Manure	organic fertilizer - full dose of cattle manure
4.	Trianum P	preparation – without added fertilizers
5.	Trianum P + ½ NPK	preparation - using half the dose of mineral fertilization (NPK)
6.	Trianum P + ½ Manure	preparation - using half the dose of organic fertilization (cattle manure)
7.	Biomega TM	preparation – without fertilizers
8.	Biomega TM + ½ NPK	preparation - using half the dose of mineral fertilization (NPK)
9.	Biomega TM + ½ Manure	preparation - using half the dose of organic fertilization (cattle manure)
10.	Pisum forecrop	without fertilizers
11.	Pisum forecrop + ½ NPK	using half the dose of mineral fertilization (NPK)
12.	Pisum forecrop + ½ Manure	using half the dose of organic fertilization (cattle manure)

The initial growth of the plants, from sowing to the to the 7/8 leaf stage, was carried out in the garden in order to make the experiment as close to natural conditions as possible. For the same reason, maize grains were sown in soil brought from the field of the Agricultural Experimental Station - Złotniki branch. Soil for factor levels 1–9 was collected after rapeseed cultivation. Soil for factor levels 10–12, was collected after growing pisum of the ‘Tarchalska’ variety from Danko. According to the producer, this is a variety that achieves 108% of the standard in both dry and wet years. It is a narrow-leafed, white-flowered variety, characterized by high resistance to pod cracking and seed shedding [36]. Before pisum, wheat was grown on this site.

For incubation experiment the soil taken from the topsoil (0–25 cm) of agricultural land was used. According to the WRB classification [37] the soil was classified as Luvisol. The obtained soil material was characterized by the following content of particle size fractions: sand (2.0–0.05 mm) 51.96%, silt (0.05–0.002 mm) 42.76% and clay (<0.002 mm) 5.28%. According to USDA classification [38] the soil material was sandy loam. Soil particle-size distribution was determined with Malvern Instruments Mastersizer 2000 analyser, equipped with dispersing device Hydro 2000MU. The content of selected macronutrients and pH of soil are presented in the Table 2. The P_2_O_5_ content was assessed using the spectrophotometric method according to PN-R-04023:1996; K_2_O content, using the FEAS method according to PB-1 Ed. 1 of February 20, 2013; Mg con-tent, using the FAAS method according to PN-R-04020:1994 + Az1:2004, pH was assessed using the potentiometric method according to PN-EN ISO 10390:2022-09), and the organic C content, using the Tiurin titration method according to PB-4 Ed. 2 of February 6, 2017.

**Table 2:** Chemical properties of the soil.

Soil	P_2_O_5_ (mg/100 g)	K_2_O (mg/100 g)	Mg (mg/100 g)	pH in 1M KCl	C org (%)
Factor levels 1–9	28.1	14.5	6.7	6.7	0.69
Factor levels 10–12	24.5	18.3	9.5	6.8	0.65

Pots with a capacity of 5.6 liters and height and diagonal 25 cm were used, each filled with 5 liters of soil. The plastic pots were selected to allow for free growth of the roots and above-ground parts of the maize plants, and to measure soil respiration. Immediately after filling the pots with soil various combinations of fertilization were used. Fertilizer doses were calculated according to the expected maize yield at the site of the fields from which the soil was used. The doses of fertilizers and biological preparations were previously measured in the laboratory using a RADWAG scale. PS 600. R2 (Radom, Poland). Table 3 shows the characteristics and doses of fertilizers and preparations used. After mixing the fertilizers with the soil, 5 maize seeds of the ‘Farmoritz’ variety were sown in each pot, and the pots were placed in a random arrangement in the garden of the Department of Agronomy. The sowing date was set for the May (24.05.2023), in accordance with the recommendations for Greater Poland. On the day of sowing, the first dose of microbiological preparations was applied and then re-peated after 34 days (27.06.2023). The date and dose of microbiological preparations application was selected according to the manufacturer’s instructions and weather monitoring, so that the temperature did not drop below 5°C. In order to investigate the possibility of using the impact of plant preparations of bacterial and fungal origin on maize plants exposed to drought stress, biological preparations called Trianum-P and Biomega^TM^ were used. The first of the mentioned agents is a biological fungicide created by Koppert, used in the form of water-soluble granules, containing live spores of the fungus *Trichoderma harzianum* T-22 at a concentration of 1x10⁸ cfu/gram. Another preparation whose effectiveness was analyzed in the experiment was a biostimulator called Biomega. This product contains strains of *Bacillus velezensis* bacteria. Both preparations were applied to the soil, in the dose given by the manufacturer. The doses calculated per pot are given in Table 3.

**Fig 1:**
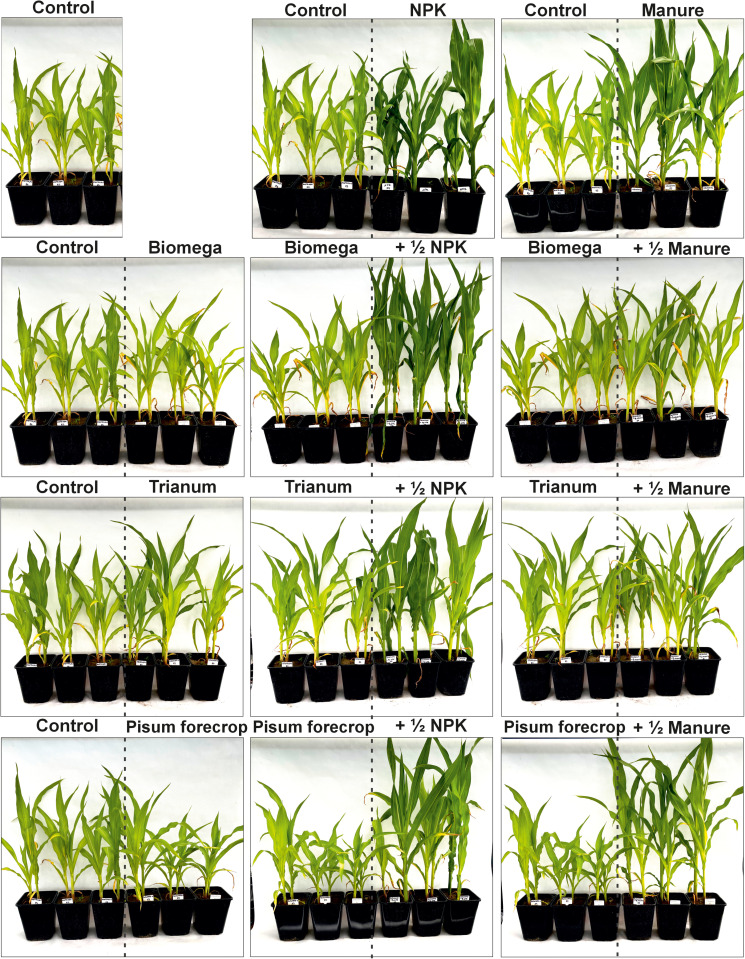
Tested levels of fertilization, microbiological preparations and forecrop under optimal water conditions.

**Fig 2:**
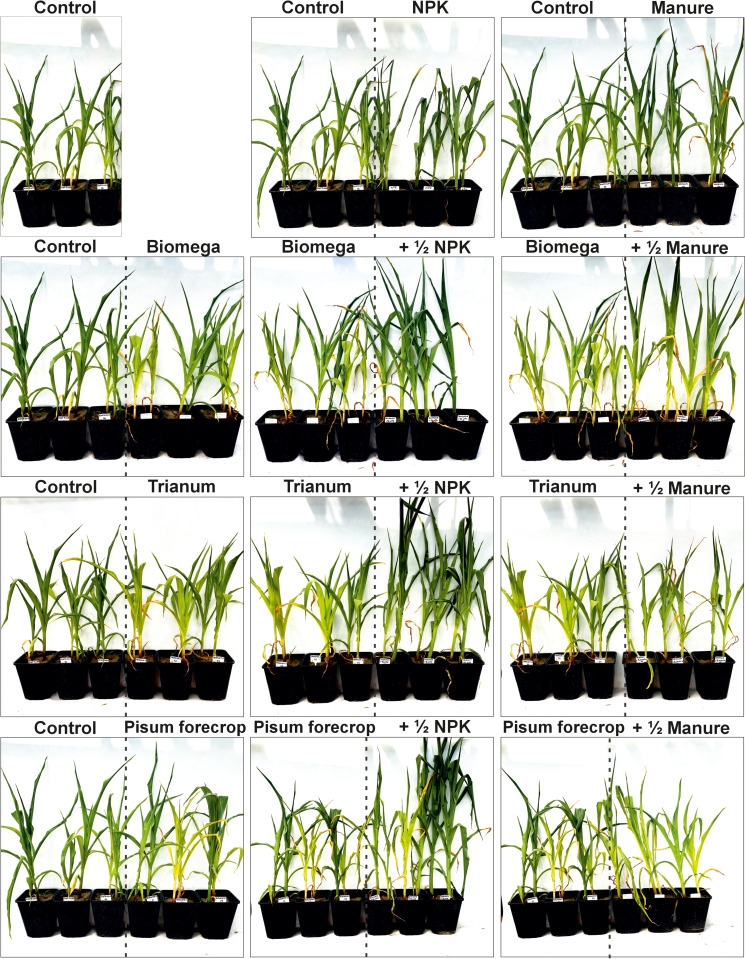
Tested levels of fertilization, microbiological preparations and forecrop under drought stress.

After 20 days from the moment of seed germination, the seedlings were discontinued and the two most uniform seedlings were left. The plants grew in the garden until they reached the 7/8 leaf stage, at which time they were moved to the greenhouse and watering was stopped in order to induce drought stress. Soil moisture was measured daily using a Geomor Technik AT W.E.T Sensor Kit moisture meter, both in stressed and watered plants. After 10 days, the soil moisture of the stressed plants reached 3 ± 1% and water became difficult to access, resulting in a stressed state. The decision to achieve a level of drought stress for maize was based on physiological traits such as saber-rolled leaves and wilting (Figs 1 and 2), as well as previous experience in finding the optimum soil moisture that induces stress but does not lead to plant death.

After inducing drought stress, the plants were transferred to the phytotron, where physiological measurements began (27.07.2023). Plants growing in optimal irrigation and under drought stress were examined alternately. All measurements were performed in 4 replicates (1 measurement for each pot), each replicate on the next day. Measurements were carried out while maintaining the same order of objects within the replicates. The planned measurements were only possible in a phytotron, which allows measurements in the complete absence of light, which is necessary to create conditions in which the only source of PAR light are the lamps of measuring devices and to make measurements when the photosynthesis process is silent. Measurements were made after 6 hours of adaptation of the plants to darkness. The temperature during the measurements was 25°C ± 1. The physiological condition of maize plants after the application of selected fertilizers/ microbiological preparations/forecrop was assessed both in plants that were induced with drought stress and in plants growing in optimal water conditions. Photosynthetic activity, relative chlorophyll content and chlorophyll fluorescence were measured on the same, youngest and fully developed leaf.

### Plant gas exchange

Photosynthetic activity was assessed by measuring gas exchange using the LCpro-SD apparatus, ADC BioScientific Ltd., UK, based on the following parameters: A - Photosynthesis Rate - CO_2_ assimilation rate (μmol m^-2^s^-1^), E - Transpiration Rate (mmol m^-2^s^-1^), Gs – Stomatal Conductance (mol m^-2^s^-1^). Device settings were selected for the experiment according to the manufacturer’s instructions (Instruction Manual, LCpro-SD Portable Photosynthesis System, Issue 4, 2012). The air flow supplied to the measurement chamber (u) was set at 200 µmols^-1^, the CO_2_ and H_2_O concentrations were set to ambient, i.e. the actual concentration in the surroundings. The intensity of light emitted in the measurement chamber (PPFD - photosynthetic photon flux density) by LEDs with red and blue spectral colors was set to 400 µmolm^-2^s^-1^ (LCP Narrow Lamp, ADC BioScientific Ltd., UK). A measurement of 4 plants (1 plant from 1 pot) was carried out lasting 20 minutes, with the measurement set every minute in order to obtain stable gas exchange results.

### Plant Chlorophyll Fluorescence and Chlorophyll content index

Chlorophyll Fluorescence was measured using the Fluorometer OS5p, OPTISCIENCES.INC, Hundson, USA. The following parameters were measured: F0 - initial fluorescence, Fm - maximum fluorescence, Fv/Fm - maximum photochemical efficiency of PSII, Yield - quantum yield of the photochemical reaction in PSII, ETR – electron transport rate, NPQ - Non - Photochemical Quenching. A kinetic protocol was chosen, which combines measurements in light (Yield, ETR, NPQ) with measurements of dark-adapted plants (F0, Fm, Fv/Fm). The fluorimeter settings were selected according to the manufacturer’s instructions (OS5p User’s Guide, The standard in Plant Stress Measurement, Opti-Sciences, 040113). The light modulation source was set to red with an intensity of 26 ranging from 1 to 32, where 17 corresponds to 0.1 µmol m^-2^s^-1^. The optimal setting is the highest possible intensity that does not induce variable fluorescence. Saturation Flash was set to an intensity of 30 ranging from 1 to 32, where 32 corresponds to 8550 µmols. The measurement cycle was set to two saturation pulses spaced 255 seconds apart. Similarly, to the above-mentioned measurement, 4 plants were measured (1 plant from 1 pot).

The Chlorophyll Content Index (CCI) was determined using the CCM – 200 Plus chlorophyllometer. Based on the measurement of chlorophyll absorption in the blue and red bands, the device calculates the relative chlorophyll content, which is proportional to the amount of this pigment in the examined leaf fragment. The units of the chlorophyll content and fluorescence parameters are unnominated. All the above-mentioned physiological measurements were performed in the same order/number of replicates and on the same leaf.

### Soil gas exchange

Soil respiration measurements were carried out after completing the physiological measurements, immediately after cutting the plants for biometric measurements.

Soil respiration measurements assessed based on following parameters: NCER- Net CO_2_ Exchange Rate (µmol m^-2^ s^-1^), W flux- Net H_2_O Exchange Rate (mmol m^-2^ s^-1^) and Ce- Soil Respiration (µmol s^-1^) using the LCpro-SD (ADC BioScientific Ltd., Hoddesdon, UK) device.

The device has a metal cylinder that is placed in the soil (pot), and after 30 minutes a one-liter acrylic chamber is placed on it to enclose air and measure gas exchange between the soil and the atmosphere. The chamber has a built-in fan for mixing the air and a bleed-off valve preventing the formation of an excessive pressure gradient inside the chamber. The concentration of CO_2_ supplied to the measuring soil chamber (reference CO_2_) and the concentration of H_2_O (reference H_2_O) was set to ambient (the actual concentration in the environment). The air flow to the measuring chamber (u) was maintained at 200 μmol/s. The one half-hour measurement was performed in each pot.

Soil Respiration (Net Molar Flow of CO_2_ in/out of the Soil)

Symbol: Ce (µmol s^-1^),


Ce=u(−ΔC)

Where,

u = molar air flow in mol s^-1^

∆C – difference in CO_2_ concentration through soil pot, dilution corrected, µmol mol^-1^.

Netto CO_2_ Exchange Rate (Ce per unit area)

Symbol: NCER (µmol m^-2^ s^-1^)


NCER=us(−ΔC)

Where,

us = molar flow of air per square meter of soil, mol m^-2^ s^-1^.

∆C – difference in CO_2_ concentration through soil pot, dilution corrected, µmol mol^-1^.

Net H_2_O Exchange Rate (Soil Flux)

Symbol: Wflux (m mol m^-2^ s^-1^)


$$Wflux\frac{\left(\Delta e\;us\right)}{p}$$

Where,

us – molar flow of air per square meter of soil, mol m^-2^ s^-1^

∆e – differential water vapor concentration, mbar, dilution corrected

p – atmospheric pressure, mBar

### Shoots and roots dry mass

Measurements of the dry weight of shoots (g) and roots (g) were made using a laboratory dryer Type: SLW 240 ECO (POL-EKO-AARAURA SP.J. A. Polok- Kowalska, S. Kowalski. EN ISO 9001:2008 PN-N 18001:2004) at temp. 65˚C until a constant dry mass was obtained, and RADWAG laboratory scale PS 600. R2 after completing physiological measurements, immediately after cutting the plants.

### Statistical analysis

We examined the effects of two factors -presence of drought stress and combinations of fertilization, microbiological preparation application and forecrop on the physiological status of maize seedlings using two-way ANOVA with post-hoc Tukey’s Honestly Significant Difference test at α = 0.05 using the data from four independent replicates. The normality of the distributions of the observed traits was tested using Shapiro-Wilk’s normality test (Shapiro& Wilk, 1965) to verify whether the analysis of variance (ANOVA) met the assumption that the ANOVA model residuals followed a normal distribution. Statistical analyses were performed using the GraphPad Prism software (v.8.0) combined with the IBM SPSS software (v.1.0.0.1327).

## Results

### Plant gas exchange

Significant differences were noted in the results of all three photosynthesis parameters tested. The highest CO_2_ assimilation rate was observed in conditions of optimal watering for the levels: Pisum forecrop + ½ NPK and Pisum forecrop + ½ Manure and then on the levels: Trianum + ½ NPK and Trianum + ½ Manure. In drought conditions, the most intensive CO_2_ assimilation was noted for the levels: Trianum + ½ NPK and Trianum + ½ Manure. The greatest transpiration level under optimal watering conditions was assayed at the levels: Pisum forecrop + ½ Manure, Trianum + ½ NPK and Pisum forecrop + ½ NPK, and under drought stress: Trianum + ½ Manure, Trianum + ½ NPK, Pisum forecrop + ½ NPK and Pisum forecrop + ½ Manure. The greatest stomatal conductance under optimal watering conditions was obtained at the levels: Pisum forecrop + ½ Manure, Pisum forecrop + ½ NPK, Trianum + ½ NPK and Trianum + ½ Manure, and under drought stress at the levels: Trianum + ½ NPK, Pisum forecrop + ½ Manure and Trianum + ½ Manure.

In summary, the most effective photosynthesis under optimal watering conditions was observed in maize plants where the Pisum forecrop was applied together with fertilizers, whereas in drought conditions in plants where the Trianum preparation with fertilizers were used (Table 4).

**Table 4:** Photosynthesis measurement parameters depending on the combinations of fertilization, preparation application and forecrop used under drought stress.

Treatment	A (μmol·m^ − 2^s^ − 1^)		E (mmol·m^ − 2^s^ − 1^)		Gs (mol·m^ − 2^s^ − 1^)	
	Control	Drought	Control	Drought	Control	Drought
Control	5.22 ^f^	0.08^h^	0.58^ghi^	0.33^j^	0.04^e-i^	0.01^i^
NPK	6.69 ^e^	0.41^h^	0.73^fg^	0.40^ij^	0.05^dh^	0.02^ghi^
Manure	7.30^e^	3.08^q^	0.76^fg^	0.35^j^	0.06^c-f^	0.01^i^
Biomega	4.51^f^	0.64^h^	0.67^fgh^	0.31^j^	0.05^d-g^	0.01^i^
Biomega + ½ NPK	9.15^d^	0.81^h^	1.10^de^	0.49^hij^	0.06^c-f^	0.03^f-i^
Biomega + ½ Manure	11.01^c^	1.61^h^	1.19^cde^	0.84^f^	0.07^b-e^	0.05^c-f^
Trianum	8.32d^e^	6.93^e^	1.01^e^	0.66^fgh^	0.04^d-i^	0.05^c-f^
Trianum + ½ NPK	14.28^b^	14.14^b^	1.60^a^	1.20^cde^	0.10^a^	0.09^abc^
Trianum + ½ Manure	13.95^b^	12.09^c^	1.31^bcd^	1.38^bc^	0.10^ab^	0.07^b-e^
Pisum forecrop	10.64^c^	0.54^h^	1.32^bcd^	0.29^j^	0.07^b-e^	0.02^hi^
Pisum forecrop + ½ NPK	16.48^a^	7.47^e^	1.44^ab^	1.18^cde^	0.11^a^	0.05^def^
Pisum forecrop + ½ Manure	16.06^a^	8.46^de^	1.63^a^	1.11^de^	0.11^a^	0.08^bcd^
HSD _α = 0.05_	1.210		0.163		0.021	

A - Photosynthesis Rate - CO_2_ assimilation rate, E - Transpiration Rate, Gs – Stomatal Conductance. Different letters a–j indicates statistically different mean values (α = 0.05).

### Plant Chlorophyll Fluorescence and Chlorophyll content index

Significant differences were noted in all measured chlorophyll fluorescence parameters, although the results are very diverse. In the case of the Yield parameter, the significantly greatest value was obtained for plants from the Pisum forecrop + ½ Manure level in optimal watering conditions. For the ETR parameter, the significantly highest values were also recorded in optimal watering conditions for the Pisum forecrop + ½ Manure level, but significantly high compared to the remaining levels also for: Manure, Trianum + ½ Manure, Biomega + ½ Manure and Biomega + ½ NPK. Non - Photochemical Quenching (NPQ) was quite even in values, and the most outlier and lowest result was observed in plants fertilized only with NPK under drought stress. In the case of the F0 parameter, the lowest values are the most favourable and were recorded for the levels: Manure and Biomega in optimal hydration conditions and Biomega +½ Manure in drought stress conditions, as well as Trianum in both water regimes. Significantly the greatest Fm values were found in plants from the Biomega +½ NPK, Pisum forecrop + ½ NPK, Pisum forecrop + ½ Manure, Trianum +½ NPK, NPK and Manure levels in both water regimes, as well as Trianum + ½ Manure and Pisum forecrop in optimal watering conditions. For the Fv/Fm parameter, the significantly greatest values were recorded in plants from the levels: Biomega +½ NPK and Manure in both water regimes, and in Pisum forecrop + ½ NPK, Trianum + ½ Manure, Trianum + ½ NPK and NPK in conditions of optimal irrigation.

**Fig 3:**
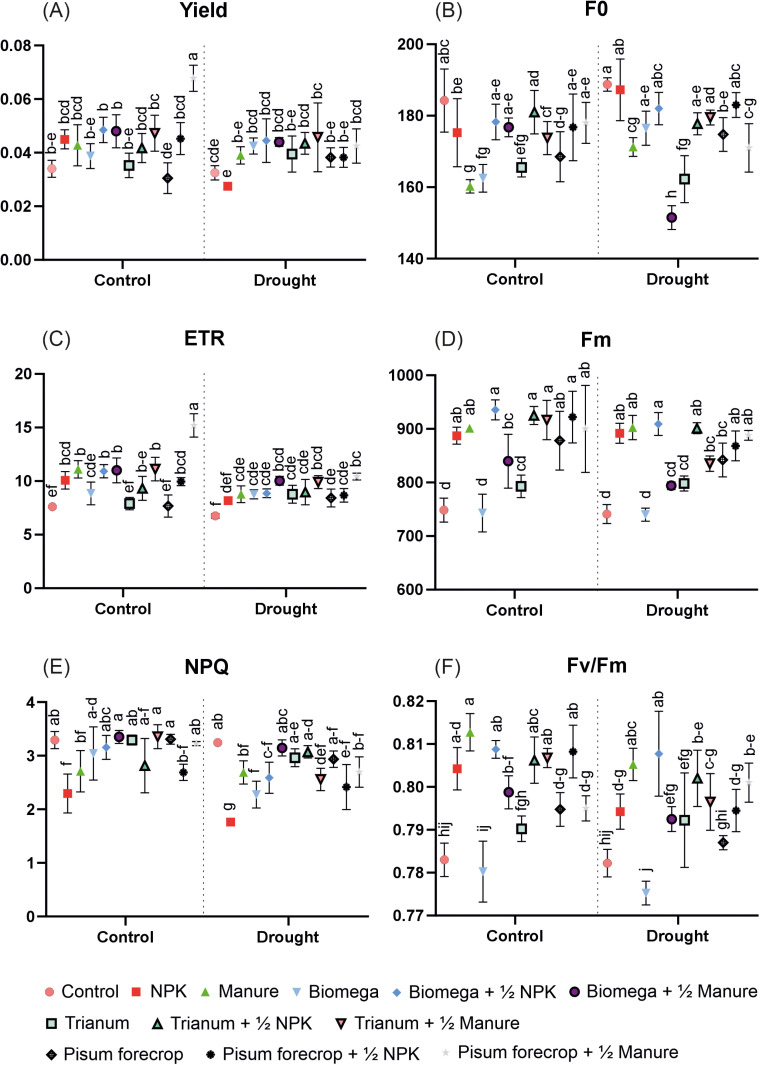
Chlorophyll fluorescence: (A) Yield - quantum yield of the photochemical reaction in PSII, (C) ETR – electron transport rate, (E) NPQ - Non - Photochemical Quenching, (B) F0 - initial fluorescence, (D) Fm - maximum fluorescence, (F) Fv/Fm - maximum photochemical efficiency of PSII. Unnominated units. Different letters a–j indicates statistically different mean values (α = 0.05). HSD α = 0.05: Yield – 0.0081, ETR – 1.12, NPQ – 0.338, F0 – 7.5, Fm – 39.6, Fv/Fm – 39.6.

Analyzing all chlorophyll fluorescence parameters, the overall greatest efficiency of photosystem II can be attributed to plants growing in optimal watering from the levels: Biomega +½ NPK, Pisum forecrop + ½ Manure, Trianum + ½ Manure and Manure (Fig 3).

**Fig 4:**
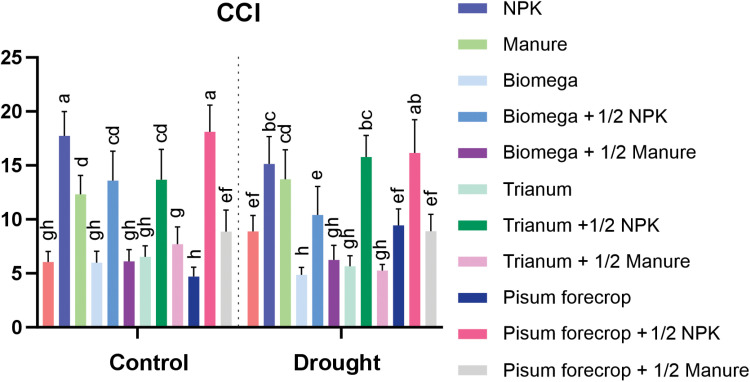
CCI - Chlorophyll Content Index. Unnominated units. Different letters a–h indicates statistically different mean values (α = 0.05). HSD α = 0.05: 1.68.

The significantly greatest chlorophyll content was found in plants growing in optimally watered conditions, as well as in drought stress at the Pisum forecrop + ½ NPK level, and in plants growing in optimally watered conditions at the NPK level (Fig 4).

### Soil gas exchange

The significantly greatest values of the NCER and Ce parameters, under optimal watering conditions, were noted at the levels: Pisum forecrop + ½ Manure, Biomega + Manure, and then: Pisum forecrop + ½ NPK and Trianum + ½ Manure, while under drought stress conditions the values were significantly distinguished only at the level of Pisum forecrop + ½ Manure. In the case of the W flux parameter, no large differences were noted, only the Control in optimal hydration conditions was characterized by a significantly outlier result of this parameter.

To sum up, the most effective soil respiration in both water regimes was observed in maize plants after the Pisum forecrop with manure was applied (Table 5).

**Table 5:** Soil Respiration measurement parameters depending on the combinations of fertilization, preparation application and forecrop used under drought stress.

Treatment	NCER (μmol·m^ − 2^s^ − 1^)		W flux (mmol·m^ − 2^s^ − 1^)		Ce (μmol·s^ − 1^)	
	Control	Drought	Control	Drought	Control	Drought
Control	2.38^gh^	2.34^gh^	0.28^c^	0.43^ab^	24.4^e-i^	22.3^i^
NPK	3.38^c-g^	2.76^e-h^	0.50^a^	0.38^abc^	32.1^c-g^	26.2^d-i^
Manure	3.70^b-e^	3.20^c-h^	0.47^ab^	0.40^abc^	35.2^bcd^	29.2^d-i^
Biomega	3.30 cg	2.52^fgh^	0.45^ab^	0.43^ab^	31.4^c-h^	23.9^f-i^
Biomega + ½ NPK	3.48^c-f^	2.45^gh^	0.45^ab^	0.41^ab^	33.1^c-f^	23.2^ghi^
Biomega + ½ Manure	4.63^a^	3.11^d-h^	0.46^ab^	0.35^bc^	44.0^a^	29.5^d-i^
Trianum	2.77^e-h^	2.37^gh^	0.38^abc^	0.47^ab^	26.4^d-i^	32.3^c-g^
Trianum + ½ NPK	3.19^c-h^	2.40^gh^	0.47^ab^	0.40^abc^	33.7^cde^	22.8^hi^
Trianum + ½ Manure	4.09^a-d^	3.33^c-g^	0.48^ab^	0.39^abc^	41.2^ab^	31.6^c-h^
Pisum forecrop	3.51^c-f^	2.21^h^	0.41^ab^	0.38^abc^	33.3^cde^	21.0^i^
Pisum forecrop + ½ NPK	4.13^abc^	2.78^e-h^	0.43^ab^	0.38^abc^	39.2^abc^	26.4^d-i^
Pisum forecrop + ½ Manure	4.77^a^	4.42^ab^	0.42^ab^	0.37^abc^	45.3^a^	42.0^ab^
HSD _α = 0.05_	0.595		0.071		5.37	

NCER- Net CO_2_ Exchange Rate, W flux- Net H_2_O Exchange Rate and Ce- Soil Respiration.

Different letters a–i indicates statistically different mean values (α = 0.05).

### Plants and roots dry mass

Significant differences were noted between the applied levels of fertilization and the preparations/forecrop used, both in shoot and root dry mass (DM). In optimal watering conditions, the greatest shoot dry mass was obtained at the levels: Biomega + ½ NPK and then: Trianum + ½ NPK, Pisum forecrop + ½ Manure and Manure, Trianum + ½ Manure, Pisum forecrop + ½ NPK, while the greatest root dry mass was obtained at the same levels, but in the following order: Pisum forecrop + ½ Manure, Biomega + ½ NPK, Trianum + ½ Manure, Trianum + ½ NPK, Pisum forecrop + ½ NPK and Manure. In turn, under drought stress conditions, the greatest shoot dry mass was obtained at the levels: Pisum forecrop + ½ NPK, Trianum + ½ NPK and Biomega + ½ NPK, while the greatest root dry mass was obtained at the levels: Pisum forecrop + ½ NPK, Pisum forecrop + ½ Manure, Trianum + ½ NPK, Biomega + Manure and Biomega + ½ NPK.

To sum up, in optimally watered conditions the highest DM shoots was observed in plants where the Biomega and Trianum preparations with NPK were used, while in drought conditions in plants after the Pisum forecrop with NPK. In turn, the largest roots were found in plants after the Pisum forecrop, in control conditions with NPK fertilization and in drought conditions with manure (Table 6).

**Table 6:** Dry mass of shoots (g) and roots (g) depending on the combinations of fertilization, preparation application and forecrop used under drought stress.

Treatment	Shoot DM (g)		Root DM (g)	
	Control	Drought	Control	Drought
Control	10.2^gh^	10.0^gh^	8.26^e-h^	6.98^fgh^
NPK	16.7^c-h^	20.0^b-e^	6.38^gh^	9.69^c-h^
Manure	21.9^b-e^	16.8^c-h^	13.56^b-f^	9.72^c-h^
Biomega	10.9^fgh^	10.7^fgh^	9.07^d-h^	9.79^c-h^
Biomega + ½ NPK	26.6^b^	24.4^bc^	15.64^bcd^	12.59^b-g^
Biomega + ½ Manure	17.7^c-g^	10.7^fgh^	9.88^c-h^	15.35^b-e^
Trianum	12.9^e-h^	13.4^e-h^	8.31^e-h^	9.62^c-h^
Trianum + ½ NPK	23.3^bcd^	26.7^b^	14.87^b-e^	15.75^bcd^
Trianum + ½ Manure	20.4^b-e^	14.6^d-h^	14.90^b-e^	11.18^c-h^
Pisum forecrop	8.1^h^	13.6^e-h^	5.40^h^	9.99^c-h^
Pisum forecrop + ½ NPK	21.7^b-e^	38.0^a^	14.68^b-e^	23.01^a^
Pisum forecrop + ½ Manure	22.8^bcd^	17.2^c-g^	16.75^bc^	18.74^b^
HSD _α = 0.05_	5.20		4.009	

Different letters a–h indicates statistically different mean values (α = 0.05).

## Discussion

### Soil gas exchange

The plants yield could be adversely influenced by the shortage of water supply from the soil, limitations in nutrients uptake and reduction in photosynthetic rate [39]. It is also known that many of soil bacteria and fungi are involved in fixing nutrients in the soil [11]. The most common application of this role is intercropping or crop rotation, especially legumes, which increase the population of nitrogen-fixing bacteria in the soil [40]. Microorganisms decompose organic remains into humus, which contains nutrients that plants need for growth, such as ammonium ions, which is produced during the decomposition of dead matter [11]. Additionally, using an organic fertilization promotes a higher soil microorganism diversity under drought [39]. It seems that the key to success, here was the combination of organic fertilizer with microorganisms and Pisum forecrop. The significant greatest values for both parameters (NCER and Ce), were noted for plants in which manure was used in combination with a Pisum forecrop in both water regimes, and with preparations based on Trichoderma and Bacillus, or even manure alone in optimal water condition (Table 5). Under drought stress conditions, the highest netto increase on CO_2_ exchange rate was noted in the case of using manure with the Pisum forecrop (89%). A similar trend was noted in the aspect of soil respiration, the increase in Ce under drought stress conditions was as much as 88% in the combination of manure with the Pisum forecrop. Additionally, in the case of this parameter, a significant increase was noted in plants treated with Trianum alone and with manure (Table 5). Similarly, Jurys and Feizienė [40], using biological preparations based on Bacillus and Trichoderma, noted an increase in soil respiration by 17 and 16%, respectively (average of two seasons). The increase in CO_2_ emission and soil respiration is directly related to the greater activity and microbiological biodiversity of the soil. Regardless to kind of fertilization, the increased biodiversity of soil resulted from microbiological preparations application as well as Pisum forecrop induce the greater root mass of maize (Table 6) which also rose the CO_2_ emission. According to Sun et al. [35] studies, the greatest soil respiration was also achieved with organic fertilization, which provides a greater diversity of bacteria in drought conditions than mineral fertilization. According to the authors, in an organically fertilized environment, there is a greater resistance of microorganisms to the depletion of energy for adaptation to drought, and a better regeneration of bacterial communities than in soil where only mineral fertilization was applied. The introduction of beneficial soil microorganisms such as Bacillus and Trichoderma positively affects the native soil microbiota by increasing microbial diversity and activity, and thus drought tolerance [41]. Therefore, the use of bioproducts supports the soil through many complex aspects, increasing the biodiversity of microorganisms, fixing nitrogen, dissolving phosphorus, excreting phytohormones, or even producing substances that suppress phytopathogens, and thus improves the protection of plants against abiotic and biotic stress [41].

### Plants and roots dry mass

Our results indicate that the microorganisms and forecrop used in the experiment could compensate the effects of drought and improved plant growth and development due to better absorption of water and nutrients from the soil. However, the use of microbiological preparations or the Pisum forecrop alone is not enough, only the use of microbiological preparations and the forecrop with the half fertilizer dose gave a synergistic effect of improving DM. At optimal irrigation and reduced NPK dose, plants with microbiological preparations/forecrop used were characterized by higher DM of shoots than plants where the full dose of fertilizer, without microbiological preparations, was applied. In the case of drought stress, this tendency is also visible, but significant difference was proven only for the Pisum forecrop. Taking into account the use of manure, the results do not indicate significant differences in both water regimes (Table 6). The use of microbiological preparations and forecrop with the half of NPK dose significantly rose root DM in both water regimes. In the case of manure application, a significant increase in the root DM despite the reduction of the fertilizer dose by half was observed only in the case of the use of the Biomega preparation and the Pisum forecrop in drought conditions. In the studies of Fonseca et al. [42] revealed that application of Bacillus subtilis inoculation improved plant root growth (length and mass) only in drought conditions. In the study by Romero-Munar et al. [43], the increase in dry matter in drought conditions was greater than in the control conditions. Similarly, to the other studies [39,44–47] we observed that in comparison to control object without microorganisms, the application of both microbiological preparations with half dose of NPK resulted in higher DM of shoots and roots in both water regimes (Table 6). Also, Harmann et al. [18] indicated that Trichoderma increased root development and the proliferation of secondary roots as well as seedling fresh weight and foliar area. In turn, the studies by Tiepo et al. [33] pointed increased dry mass of roots and shoots in maize plants as a result of inoculation with Bacillus velezensis. This effect may be related to the influence of both PGPB (Plant Growth-Promoting Bacteria) and AMF (Arbuscular Mycorrhizal Fungi) on the production of auxins, which stimulate root development and plant growth [19,33]. Under drought conditions, the highest increase in shoots dry mass compared to the drought control was 280%, 167% and 144%, respectively, in plants treated with the forecrop Pisum with NPK, Trianum with NPK and Biomega with NPK. In comparison, the use of NPK alone without a microbiological preparations increased the shoots DM by 100% (Table 6). While the highest increase of roots DM was recorded in the combinations of Pisum with NPK, Pisum with Manure, Trianum with NPK and Biomega with manure, by 230%, 168%, 126% and 120%, respectively. The mode of action of microbiological preparations improving drought tolerance could be attributed to regulating hydraulic under drought stress as a result of the increase in nitrogen content, as well as ability to solubilize P + and K+ under stress conditions [48]. The research of Moreno-Galván et al. [47] revealed that inoculation with Bacillus spp. increased the uptake of K + and P + by maize plants subjected to drought stress by an average of 14% and 25% for 5 strains. According to Matias et al. [49], fungal hyphae can take up phosphorus and ammonium ions much more efficiently than plant roots. Moreover, this effect may be attributed due to the synthesis of components responsible for stimulating root growth, including indole-3-acetic acid (IAA), gibberellins and cytokinins by the microorganisms [46]. Similar mechanisms underlie the improvement in drought stress tolerance following the use of Pisum forecrop. Legume species in crop rotation have a positive effect on soil biological activity by increasing the organic carbon content in the soil [50], humus content and activity of soil microorganisms [51] as well as on soil physical properties, affecting aggregate stability, improving infiltration coefficients and hydraulic conductivity [52]. Thanks to their nitrogen fixation activity, the legume species improves soil N content [51,53], and consequently reduces N fertilization needs in the next crop [54].

### Plant gas exchange

The negative anatomical effects resulting from water deficiency in the soil and limitations in nutrient uptake correspond directly to the reduction in photosynthetic efficiency. Reduced water flow from the roots affects stomatal conductance, transpiration and photosynthetic rate through limited gas exchange [55]. The synergistic effect of irrigation and nitrogen fertilization significantly affects photosynthetic parameters and carbon metabolism processes in maize plants. Optimally planning fertilization adequately to water resources may increase the activity of key enzymes, such as sucrose phosphate synthase and phosphoenolpyruvate carboxylase. This increased enzymatic activity increases sucrose synthesis and carbon dioxide fixation efficiency [56]. An improvement in photosynthetic efficiency expressed as CO_2_ uptake intensity was noted in almost all combinations compared to the control, both under optimal watering and drought stress conditions (Table 4). The greatest, significant effect in optimal watering conditions was visible in plants with the Pisum forecrop, where the increase in CO_2_ assimilation efficiency was as much as 55% (Pisum forecrop with NPK) and 51% (Pisum forecrop with Manure) compared to the control after the Pisum forecrop, while in comparison to the Control, it was 216% and 208%, respectively. A significant increase in CO_2_ assimilation efficiency was also noted in plants, when using the Trichoderma preparation, the increase was 174% (Trianum with NPK) and 167% (Trianum with Manure). A significant increase in photosynthesis efficiency was also noted in plants using the Bacillus preparation, but it was not as spectacular. In turn, the measurement of photosynthesis efficiency under drought stress conditions clearly indicated the highest, significant effect after the application of the Trichoderma preparation with both fertilizers. In the control, CO_2_ assimilation was practically inhibited due to drought (98% drop), while in the plants in which the Trichoderma preparation was used together with half dose of NPK and manure, there was only a slight decrease (1% and 13% respectively). According to Vargas et al. [20], Trichoderma virens induced higher photosynthetic efficiency by increasing CO_2_ uptake by leaves. A significant improvement in photosynthetic efficiency in drought was also noted for maize plants after the forecrop with Pisum, both with NPK and Manure (Table 4). A similar trend was noted for the remaining parameters, transpiration and stomatal conductance. The highest, significant level of water evaporation and stomatal conductance was obtained for plants in which the Pisum forecrop and the Trianum preparation were used in both fertilization combinations (Table 4). In the work of Da Silva et al. [57], soybean plants inoculated with *Bacillus amyloliquefaciens* and *Trichoderma asperellum* also maintained high photosynthetic rates without changing stomatal conductance under drought stress, which allowed for greater carbohydrate acquisition without tissue dehydration. Drought stress can reduce photosynthetic activity by causing stomatal closure and consequently lowering gas exchange and CO_2_ assimilation. It leads to the impairment of the photosynthetic apparatus due to the imbalance in energy supply and demand. Reduction in CO_2_ fixation in the Calvin Benson cycle increases electron accumulation in photosystems I and II, resulting in greater ROS (Reactive Oxygen Species) generation and lipid peroxidation [55]. According to Tiepo et al. [34], the inoculated plants maintained a positive carbon balance, and drought did not cause oxidative stress, as the plants avoided ROS formation, probably through alternative electron pathways [57]. Other researchers also observed significant improvement in the efficiency of photosynthesis and transpiration under drought stress in maize plants after the application of Trichoderma [58] and Bacillus support [43,59]. This trend is also true for other plant species. Fonseca et al. [42] reported a significant increase in photosynthetic activity under both drought stress and optimal water conditions as a result of the use of Bacillus in Sugarcane, while Bae et al. [39] noted similar positive effect for Trichoderma inoculation in cacao. Fonseca et al. [42] drew similar conclusions that the preservation of photosynthetic activity despite drought is related to the protective role of microorganisms against chlorophyll degradation by ROS.

What is most important, despite drought stress and reduction of the manure dose by half, all three photosynthesis parameters significantly increased as a result of the use of microbiological preparations and the Pisum forecrop. In the case of NPK dose reduction in drought stress, no significant improvement in photosynthesis efficiency was noted only in the case of the use of Bacillus, although such a tendency is visible (Table 4). According to the above-cited study by Huang et al., treatments increasing water availability in maize plants allow for a reduction in the fertilization dose without negative effects on the yield, and even allow for achieving higher yields thanks to increased photosynthesis efficiency [56].

Water limitation causes redox imbalance in cells due to increased production and accumulation of reactive oxygen species (ROS) and can lead to lipid peroxidation, photooxidation of pigments and chlorophyll degradation and consequently to reduced photosynthetic efficiency. Recent studies indicate a role of melatonin in the direct removal of reactive oxygen species by increasing the activity of antioxidant enzymes, as well as by regulating stress-related transcription factors [60]. Melatonin, in addition to its antioxidant properties, is known for its potential to stimulate plant growth, acts as a versatile phytohormone, maintaining metabolic balance under stressful conditions, promoting nitrogen assimilation and protects plants from abiotic stress by regulating photosynthesis [61]. Melatonin is believed to be produced by plant growth-promoting bacteria (PGPB) including *Bacillus amyloliquefaciens*. B. velezensis is a later heterotypic synonym of B. amyloliquefaciens based on DNA-DNA relatedness values [62]. The increased efficiency of photosynthesis through the use of microorganisms could have been due to the protective effect of melatonin. However, this assumption needs to be explored in further research.

### Plant Chlorophyll Fluorescence and Chlorophyll content index

The strategy to improve photosynthetic activity by reducing ROS concentrations in plant tissues affected by drought stress is supported by the results of chlorophyll fluorescence in our studies (Fig 3). In other studies, on wheat biostimulation with various preparations, the preparation containing 5 strains of soil bacteria of the genus Bacillus showed the greatest protection against drought, which was also proven by the highest photosynthetic efficiency and the lowest degradation of the photosynthetic apparatus determined by measuring chlorophyll fluorescence [63]. The Fv/Fm parameter, which determines the potential efficiency of PSII and can be used as an indicator of the photochemical activity of the photosynthetic apparatus of the tested plants [64], was the significantly greatest in both water regimes in plants treated with preparations containing Bacillus, Trichoderma with NPK and with manure alone (Fig 3F). In other studies, Fv/Fm also retained the highest values under drought conditions in plants treated with Bacillus [43,48,59,65] and Trichoderma [58] preparations. The Yield parameter (Fig 3A) in-forms about the ratio of the quantum of light excitation necessary for photochemical transformations to the total number of absorbed photosynthetically active radiation (PAR), while the ETR parameter (Fig 3B) evaluates the rate of electron flow through photosystems. The values of these parameters decrease as a result of damage or reduced efficiency of the photosynthetic apparatus, e.g. due to drought [66]. The Yield parameter, retained the significant greatest values in drought conditions in plants treated with preparations containing Bacillus and Trichoderma with both fertilizers and plants after the Pisum forecrop in combination with manure. In the case of electron transport rate (ETR), significantly higher results comparing the control object, were recorded for all combinations except NPK, but the highest values were demonstrated for microbiological preparations and the Pisum forecrop in combination with manure (Fig 3B).

## Conclusions

Microbiological preparation containing Trichoderma fungi, as well as the Pisum forecrop enable to reduce the fertilization dose by at least half, without a negative impact and even with a benefit for maize plants. The positive effects of those factors refer to physiological condition of plants expressed through the efficiency of photosynthesis and the dry mass of roots as well as soil respiration. There was also noted positive influence of Bacillus preparation and half fertilization dose combination however it was not statistically proven, which requires further investigation, preferably under field conditions.

The greatest improvement in soil gas exchange for both water regimes was demonstrated in the case of maize plants in which the Pisum forecrop was applied together with half manure dose. A significant improvement in the dry mass of roots in the same objects, and in plants in which the forecrop was applied together with half NPK dose, confirms that these factors provide the most favorable conditions for the roots, which is particularly important in drought stress.

The measurement of photosynthesis efficiency under drought stress clearly indicated the greatest, significant effect caused by Trichoderma preparation with half dose of both fertilizers. In the optimal watering conditions, the most positive effect on maize photosynthesis efficiency had Pisum forecrop applied together with half dose of fertilizers.

Fertilization is one of the largest component of maize production costs, at current prices it ranges 500–1000 euros per hectare. The 50% reduction of fertilizers demand allowed by microbal preparations application, at a level of 56 euros (Trianum) to 95 euros (Biomega), enable to reduce the costs of fertilization by 30–45%. The potential reduction of fertilizers could bring ecological and economic benefits however this assumption require research continuation especially in a field multi-year experiment which include different maize varieties and different types of soil.

The simultaneous use of microbiological preparations, balanced fertilization and a favorable forecrop can be an important basis for ensuring sustainable plant production, especially in the periods of increasing drought stress.
